# Effectiveness of a Walking Football Program for Middle-Aged and Older Men With Type 2 Diabetes: Protocol for a Randomized Controlled Trial

**DOI:** 10.2196/28554

**Published:** 2021-11-03

**Authors:** Ana Barbosa, João Brito, Pedro Figueiredo, André Seabra, Romeu Mendes

**Affiliations:** 1 EPIUnit – Instituto de Saúde Pública Universidade do Porto Porto Portugal; 2 Portugal Football School Portuguese Football Federation Oeiras Portugal; 3 Research Center in Sports Sciences, Health Sciences and Human Development (CIDESD) University Institute of Maia (ISMAI) Maia Portugal; 4 Research Centre in Physical Activity, Health and Leisure (CIAFEL) Faculdade de Desporto Universidade do Porto Porto Portugal; 5 ACES Douro I - Marão e Douro Norte, Northern Region Health Administration Porto Portugal

**Keywords:** type 2 diabetes, cardiovascular risk factors, physical activity, exercise, football, soccer, walking, randomized controlled trial

## Abstract

**Background:**

Studies on walking football have found positive effects on health; however, there are still several research gaps when applying walking football programs for patients with type 2 diabetes.

**Objective:**

This study aims to test the effectiveness of a walking football exercise program on glycemic control and cardiovascular risk factors in middle-aged and older men with type 2 diabetes.

**Methods:**

The study will be run as a randomized controlled trial with a 6-month duration in Portugal. Eligible participants will be randomized using a 1:1 ratio for intervention or control groups and compared using an intention-to-treat analysis. The intervention will consist of a walking football exercise program. The control group will continue with usual care in primary health care units. The primary outcome will be the mean difference in glycated hemoglobin between intervention and control groups after 6 months. Secondary outcomes include the mean differences in fasting blood glucose, total cholesterol, low-density lipoprotein cholesterol, high-density lipoprotein cholesterol, triglycerides, systolic and diastolic blood pressure, body mass index, waist circumference, fat-free mass, and fat mass. Additionally, secondary outcomes include the incidence of exercise-related injuries and adverse events and the walking football exercise program’s cost-utility.

**Results:**

The study protocol is being prepared to be submitted to the Health Ethics Committee of the Northern Regional Health Administration, Portugal. After approval, participant recruitment will start in primary health care units in Porto's metropolitan area by family medicine doctors.

**Conclusions:**

Walking football might have the potential to be effective in improving glycemic control and cardiovascular risk factors, with a low rate of exercise-related injuries and adverse events and a good cost-utility ratio. Therefore, walking football may be a sustainable intervention strategy for type 2 diabetes management.

**International Registered Report Identifier (IRRID):**

PRR1-10.2196/28554

## Introduction

### Background and Rationale

Type 2 diabetes (T2D) is a global public health concern considering its morbidity, mortality, and health expenditure [1]. In 2019, it was estimated that 463 million adults (20-79 years old) worldwide were diagnosed with diabetes, corresponding to 9.0% of all adults in this age group [1] and representing nearly 90% of T2D cases. Increased exposure to environmental factors, such as obesity and physical inactivity, has been associated with the alarming increase in the prevalence of diabetes [1]. Portugal is one of the European countries with the highest prevalence of diabetes. In 2015, the prevalence of diabetes in the adult population (25-74 years old) was 9.9% [2]. The prevalence was higher in men than in women (12.1% vs 7.8%), and higher in individuals aged 65-74 years (23.8%) compared with younger individuals [2].

The benefits of physical activity in the prevention and control of T2D have long been documented [3-6]; however, a considerable proportion of individuals with T2D do not adhere to the recommendations proposed by international organizations (eg, American Diabetes Association, American College of Sports Medicine) [7,8]. Data from Portugal revealed that about 60% of individuals with T2D reported not practicing any type of exercise [9], demanding interventions to increase physical activity levels in this population.

Recreational football is conducted as small-sided games, from 3 vs 3 to 7 vs 7, practiced 2-3 times per week, in sessions of 45-60 minutes. The practice of recreational football is an intermittent activity, with participants moving at slow speed, but with consecutive changes in direction, accelerations, and decelerations, leading to periods of moderate-to-vigorous intensity. This intermittent activity has shown cardiovascular, metabolic, and neuromuscular benefits across different populations [10-14]. This can contribute to increased physical activity levels and, therefore, to the control of several noncommunicable diseases, including T2D [10-14].

Exercise-related injuries and the high exercise intensities observed in recreational football led some football clubs to develop walking football strategies for their older players [15]. Walking football follows football’s general rules, but it does not allow players to run or have physical contact, and the ball must always be played below the players’ average waist height [16]. Available studies on walking football have reported engagement and satisfaction with the modality, the pattern of exercise intensity (from light to vigorous), and health benefits (namely on body composition, aerobic fitness, blood pressure, cognitive function, psychologic well-being, and quality of life) [17-22].

Only 3 studies provided details regarding the participants’ medical conditions [17,20,22]. Participants were mainly middle-aged and older men with overweight, obesity, hypertension, or T2D. Characterization of medical conditions and cardiovascular risk factors seems particularly important when extrapolating exercise effects for some populations. Patients with T2D have an increased risk of injuries and acute adverse events associated with exercise training compared with healthy subjects [23]. Indeed, efforts for safety are fundamental in exercise programs and may compromise participants’ adherence.

A 12-week study that tested the feasibility and safety of a walking football program in middle-aged and older men with T2D in a quasiexperimental design found that the most common adverse events were falls and musculoskeletal injuries, and no acute metabolic or hemodynamic adverse events were observed. No registered injuries or adverse events were reported, mainly due to the safety protocols applied before, during, and after each exercise session [22].

Studies on walking football showed positive effects on health. However, there are still several research gaps regarding walking football in patients with T2D. It includes limitations in study designs, sample sizes, length of the programs, assessment of variables that may influence adherence to the programs (such as the enjoyment), and impact on glycemic control and cardiovascular risk factors.

### Objectives

This study aims to test the effectiveness of a 6-month walking football exercise program on glycemic control and cardiovascular risk factors in middle-aged and older men with T2D.

This study will be accomplished through the following specific objectives: (1) evaluate the effects of a walking football exercise program on glycemic control, blood lipid profile, blood pressure, anthropometric profile, and body composition; (2) assess exercise-related injuries and adverse events of a walking football exercise program; (3) assess the cost-utility of a walking football exercise program.

## Methods

This protocol follows the Standard Protocol Items: Recommendations for Interventional Trials (SPIRIT) statement [24]. The SPIRIT checklist is available in Multimedia Appendix 1, and the trial for this protocol will be registered at ClinicalTrials.gov.

### Design

**Figure 1:**
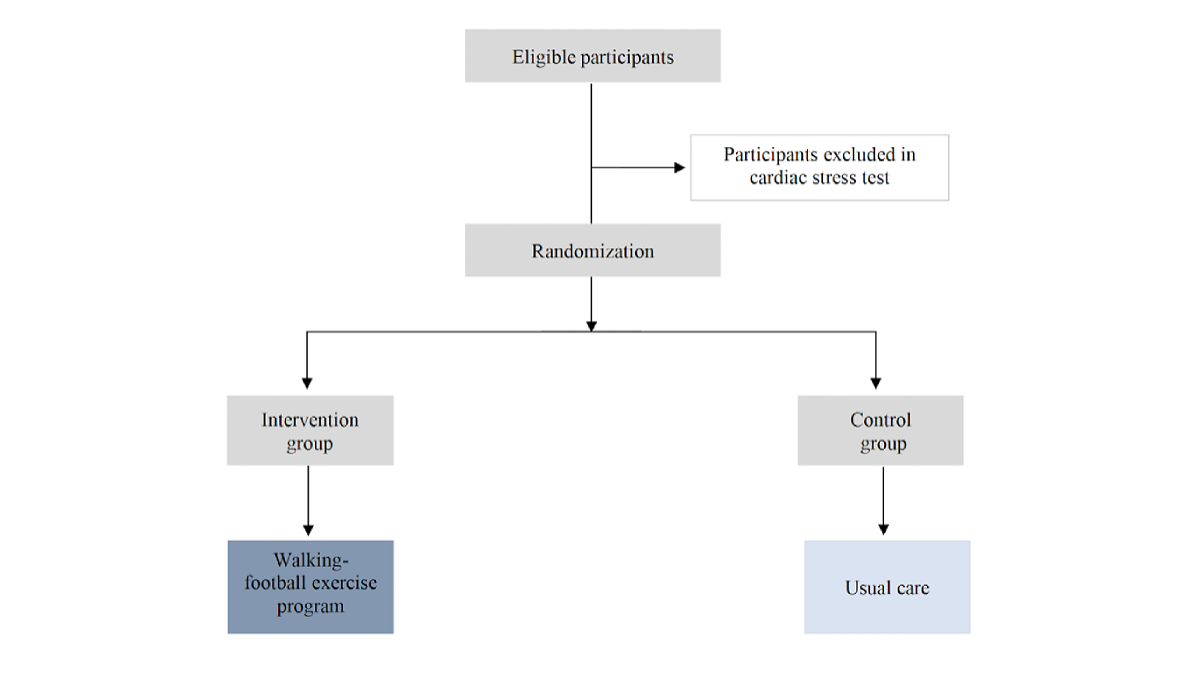
Flow diagram of the randomized controlled trial.

This study is based on a parallel-group, randomized controlled trial with a 6-month duration. Eligible participants will be randomized using a 1:1 ratio within each primary health care unit (PHCU) to intervention or control groups. The intervention will consist of a 60-minute walking football exercise program, 3 times per week (nonconsecutive days), for 24 weeks. The control group will maintain daily life routines and continue with usual care. The study design is represented in Figure 1.

### Setting

The study will be conducted in PHCU of Porto's metropolitan area, in the northern region of Portugal. A PHCU consists of multiprofessional teams, with a mean of 7 family medicine doctors, an equal number of family nurses, and administrative professionals. Family medicine doctors have a patient list that ranges from 1500 to 2000 patients, handling preventive activities and most of the acute and chronic health problems of the individuals. In these units, patients with chronic conditions, such as T2D, have regular consultations and close contact with their family medicine doctor.

### Participants

Participants will be recruited from 5 PHCUs. Family medicine doctors at these PHCUs will extract a list of potential participants from the information system and contact them by telephone. Each PHCU is expected to enroll 40 patients, corresponding to a total of 200 participants.

#### Inclusion Criteria

The participants will be selected according to the following criteria: diagnosis of T2D for at least 12 months; male; aged 55-70 years; glycated hemoglobin between 6.0% and 10.0%; not having started insulin therapy in the previous 6 months and/or sulfonylureas therapy in the previous 3 months; major complications of diabetes screened and controlled (diabetic retinopathy, diabetic nephropathy, and diabetic foot); no cardiovascular, respiratory, nor musculoskeletal contraindications to exercise; without symptoms of coronary artery disease; without limitations in gait or balance; nonsmokers at least for 6 months; not practicing supervised exercise for at least 6 months; independent living in the community; and availability for the exercise session schedule. Participants who fulfil the inclusion criteria will be invited to participate in the study and perform a treadmill cardiac stress test.

#### Exclusion Criteria

Individuals with issues identified in the cardiac stress test, namely asymptomatic cardiac or hemodynamic problems, will be excluded from the study.

### Intervention and Control Groups

Participants assigned to the intervention group will enroll in a walking football exercise program and receive basic sports material (eg, sports bag, t-shirt, and sports shoes). The participants will be organized into 5 groups of 20 players in different time schedules. Each group will have 60-minute walking football exercise sessions, 3 times per week (nonconsecutive days), for 24 weeks (72 sessions).

Walking football sessions will be conducted on a football field and supervised by a football coach certified by the Union of European Football Associations and by a nurse.

The sessions will consist of strength and conditioning exercises, technical skill drills, and small-sided and conditioned walking football games, including warm-up and cool-down periods.

The participants from the intervention and control groups will be asked to maintain daily life routines (lifestyle-related physical activity and dietary pattern) and continue with usual care (diabetes consultations and pharmacological regimen). In Portugal, the usual care at a PHCU already includes brief counseling for physical activity and sedentary behavior [25].

Participants from the control group will also receive basic sports material (eg, sports bag, t-shirt, and sports shoes).

All activities, participation rules, project team members, and the sports facilities will be presented to participants and their families before starting the intervention.

### Outcomes

The primary outcome is the difference in the change in glycated hemoglobin level between intervention and control groups after 6 months. Secondary outcomes include changes between groups in fasting blood glucose, total cholesterol, low-density lipoprotein cholesterol, high-density lipoprotein cholesterol, triglycerides, systolic and diastolic blood pressure, body mass index, waist circumference, fat-free mass, and fat mass.

Secondary outcomes also include the incidence of exercise-related injuries and adverse events, as well as the cost-utility of the walking football exercise program.

### Assignment of Interventions

The principal investigator will use a computerized random number generator to randomize participants.

Each patient will have a unique patient study number, which will be given immediately by the randomization software (the latest version of Excel Office 365).

Family medicine doctors at each PHCU will invite patients for an appointment; then, family medicine doctors will provide information about the study, assess the availability for participation, and collect informed written consent. After consent, the participants will be invited to perform a treadmill cardiac stress test.

If still eligible after the stress test, the participants will be enrolled in the study by the research team, who will provide an opaque envelope with the codification previously generated for each participant; the participant will then open the envelope and check his allocation for the intervention or control group.

Blinding of the participants, health care professionals, and research team members will not be possible.

### Data Collection

Data collection will have 3 main time points: baseline (before starting the study), 6 months (after the study ends), and every walking football exercise session. The descriptions of the variables assessed at baseline and the 6-month evaluation are presented in Table 1.

**Table 1:** Variables assessed at baseline and at the end of the study.

Variable		Method
**Glycemic control**		
	Glycated hemoglobin	Venous blood analysis
	Fasting blood glucose	Venous blood analysis
**Blood lipid profile**		
	Total cholesterol	Venous blood analysis
	LDL^a^ cholesterol	Venous blood analysis
	HDL^b^ cholesterol	Venous blood analysis
	Triglycerides	Venous blood analysis
**Blood pressure**		
	Systolic blood pressure	Automatic digital sphygmomanometer
	Diastolic blood pressure	Automatic digital sphygmomanometer
**Anthropometric profile**		
	Body mass index	Formula
	Waist circumference	Anthropometric tape
**Body composition**		
	Fat mass	Bioelectrical impedance analysis
	Fat-free mass	Bioelectrical impedance analysis
Habitual physical activity		Global Physical Activity Questionnaire score [26]
Dietary intake		3-day food record, 24-hour dietary recall analysis
Health-related quality of life		EQ-5D questionnaire [27]
Medication (number, type, and dosage)		Form

^a^LDL: low-density lipoprotein.

^b^HDL: high-density lipoprotein.

Before and after the study, habitual physical activity, dietary intake, health-related quality of life, and regular medication will be collected to be used as control variables. We will also collect sociodemographic characteristics at baseline.

At every exercise session, objective and subjective exercise intensity and enjoyment will be recorded for control purposes.

The descriptions of the variables to be assessed before, during, and after each exercise session are detailed in Table 2. Capillary blood glucose, blood pressure, and feet wounds will be assessed before the exercise session; objective exercise intensity will be recorded during the exercise session; subjective exercise intensity and enjoyment will be assessed at the end of the exercise session; and exercise-related injuries and adverse events will be evaluated during and after the exercise session if participants report symptoms or the nurse notices any issue.

**Table 2:** Variables assessed in each exercise session.

Variable		Method
Pre-exercise capillary blood glucose		Glucometer
Pre-exercise blood pressure		Automatic digital sphygmomanometer
Pre-exercise feet wounds		Self-observation
**Exercise intensity**		
	Subjective	OMNI perceived exertion scale [28]
	Objective	Heart rate and time-motion tracking
Exercise sessions enjoyment		Physical Activity Enjoyment Scale [29]
Exercise-related injuries and adverse events		Observational/clinical/self-reported

All personnel involved in the study — nurses, nutritionists, medical doctors, football coaches, and sports scientists — will receive training before baseline assessments. This aims to standardize procedures.

Specifically, the principal investigator will provide training to the other research team members regarding the clinical assessments and forms.

The Portugal Football School of the Portuguese Football Federation will provide training to football coaches to ensure the walking football program is administered in a similar way in the different centers and according to a predefined manual.

A sports scientist will monitor, in real time, internal (heart rate [HR]) and external (eg, distance covered, number of actions) workload and apply the OMNI perceived exertion scale in all sessions.

The nurse must have training in emergency procedures, be responsible for measurements at exercise sessions, manage and record exercise-related injuries and adverse events, follow-up with participants, and refer to health care facilities if necessary.

### Procedures

Before each walking football session, the nurse present at the local sports facility will evaluate capillary blood glucose (Contour XT, Ascencia Diabetes Care, Basel, Switzerland) and blood pressure (M6 Comfort, Omron, Kyoto, Japan) for all participants. Furthermore, participants will self-observe the presence of feet wounds and report any to the nurse. These measurements aim to evaluate the baseline safety conditions before the exercise session.

The participants will be allowed to start the session only under the following conditions: (1) capillary blood glucose ≥100 and ≤300 mg/dL, (2) systolic blood pressure ≤200 mm Hg, (3) diastolic blood pressure ≤100 mm Hg, and (4) no foot wounds.

During and after sessions, capillary blood glucose, blood pressure, and feet will be evaluated if participants report related symptoms. An adverse event is considered if there are any of the following after reporting symptoms: capillary blood glucose <72 mg/dL (symptomatic hypoglycemia) or >300 mg/dL (symptomatic hyperglycemia), systolic blood pressure <100 mm Hg (symptomatic hypotension) or >160 mm Hg (symptomatic hypertension response), or foot wounds are observed [3,23]. Participants in these conditions will not return to the exercise session, and corrective measures will be applied when necessary (ie, hydration, glucose intake, rest).

Strains, sprains, and contusions will be considered musculoskeletal injuries. Falls, seizures, myalgias, headache, malaise, chest pain and discomfort, and other relevant events will be considered adverse events [22].

During the walking football program, exercise intensity will be monitored systematically in every session through HR and rating of perceived exertion (RPE). From the estimated HR reserve (HRR) [30], we will classify the exercise intensity using the method by Karvonen and Vuorimaa [31]: light intensity (30%-39% HRR), moderate intensity (40%-59% HRR), vigorous intensity (60%-89% HRR), and near-maximal to maximal intensity (≥90% HHR) [32]. During training sessions, all participants will use adjustable chest strap HR monitors, and HR will be recorded at 5-second intervals using short-range radio telemetry (Firstbeat Sports, Jyväskylä, Finland). Participants will classify subjective exercise intensity through RPE using the 11-point OMNI scale (from extremely easy [0 points] to extremely hard [10 points]) at the end of the session [28]. With this RPE scale, light intensity is considered as 3-4 points, moderate intensity as 5-6 points, vigorous intensity as 7-8 points, and near-maximal to maximal intensity as 9-10 points.

### Data Analysis and Sample Size

The number of participants to be involved was defined to test the superiority of walking football compared with usual care, using intention-to-treat analysis. For a 1:1 ratio of the intervention and control groups, significance level of 5%, and statistical power of 80%, a total of 162 participants will be needed to detect a mean difference of at least 0.35% in the primary outcome (glycated hemoglobin), based on previous meta-analyses [33,34]. Assuming the complete follow-up of at least 80% of the participants, a total of 200 participants will be enrolled.

We will use a 2-way (group*time) analysis of variance with repeated measures to compare the mean differences between intervention and control groups.

For the cost-utility analysis, we will calculate the walking football implementation costs compared with the usual care. Costs include technical training for the professionals involved, patients’ medical assessments, material for capillary blood glucose and blood pressure evaluations, football equipment, sports facility rental, sports insurance, and payment to nurses and football coaches. The costs of the intervention and reported gains in quality of life (based on quality-adjusted life years) [27] will be used to calculate the incremental cost-utility ratio (ICUR). ICUR will be compared with the World Health Organization thresholds for health interventions based on per capita gross domestic product.

The amount of missing data is expected to be low considering the training of all the staff and the use of standardized procedures for data collection. No imputation is being planned.

### Ethics and Dissemination

This study will follow the General Data Protection Regulation and be submitted to the Health Ethics Committee of the Northern Regional Health Administration, Portugal. All procedures will comply with the Declaration of Helsinki. Any protocol deviation will be reported.

All participants will provide informed consent after receiving a detailed explanation of the potential risks and benefits. They will also have research insurance to cover the risks associated with exercise practice and evaluations before and after the exercise program. All participants will have a codification number to be used in the evaluations (questionnaires and blood samples). All the data collected will be treated as confidential and strictly used for this project. Data storage will be anonymized. Only the principal investigator will have access to the data.

Participants can withdraw from the study at any time, without any prejudice to the care provided at their PHCU, and have the right to access personal data collected in person from the researchers.

Findings from this study will be submitted for publication in international peer-reviewed journals. The results will also be disseminated at national and international scientific meetings and in mass media press releases.

## Results

The study protocol is being prepared to be submitted to the Health Ethics Committee of the Northern Regional Health Administration, Portugal. After approval, participant recruitment will start by family medical doctors in PHCUs in Porto’s metropolitan area.

## Discussion

### Overview

The main goal of this study is to test the effectiveness of a walking football program for glycemic control and cardiovascular risk factors in middle-aged and older male patients with T2D.

To the best of our knowledge, this is the first study testing the effects of a walking football exercise program for these outcomes in this specific population. Also, the investigation proposed relies on robust methodology, with a large study sample.

We expect that the walking football program will be effective in improving diabetes control and cardiovascular risk factors. We also expect a low rate of exercise-related injuries and adverse events and a good cost-utility ratio, as observed in other studies testing the effect of physical activity on diabetes control [35-38].

Developing an effective program with a good cost-utility ratio may contribute to making walking football a sustainable intervention strategy for T2D control. In addition, it can be used by football clubs to offer these programs as a service to their communities, ultimately contributing to the sustainability of the intervention and scaling up the offer to population coverage.

### Limitations

Some limitations need to be addressed: First, the lack of participant, health care professional, and elements of the research team’s blinding may introduce bias that may affect the outcome assessment. Nevertheless, our primary outcome is an objective measure — glycated hemoglobin, measured by blood clinical analysis — which may reduce the impact on the lack of blinding.

Second, contamination may occur since randomization units are individuals and not PHCUs, and there is proximity in the geographical area for participants from each PHCU. However, education of all research members and participants towards contamination and clear information about the purposes of the trial may minimize its occurrence.

## Appendix

Multimedia Appendix 1 The Standard Protocol Items: Recommendations for Interventional Trials checklist.

## Abbreviations

HR: heart rate
HRR: heart rate reserve
ICUR: incremental cost-utility ratio
PHCU: primary health care unit
RPE: rating of perceived exertion
SPIRIT: Standard Protocol Items: Recommendations for Interventional Trials
T2D: type 2 diabetes
